# Analysis of nursing notes and mapping of nursing surveillance activities for patients who underwent cholecystectomy in Korea: a retrospective descriptive study

**DOI:** 10.7586/jkbns.25.035

**Published:** 2025-08-22

**Authors:** Se Young Kim, Chunseon Jang, Moon Kyung Lee, Ok-Ran Jeong, Eunsim Kim

**Affiliations:** 1 Department of Nursing, Changwon National University, Changwon, Korea; 2 Edu-administration Nursing Team, Sungkyunkwan University Samsung Changwon Hospital, Changwon, Korea; 3 Rapid Response Team, Sungkyunkwan University Samsung Changwon Hospital, Changwon, Korea; 4 School of Computing, Gachon University, Seongnam, Korea

**Keywords:** Surveillance, Nursing records, Cholecystectomy, Natural language processing

## Abstract

**Purpose:**

This study aimed to systematically extract keywords from the electronic nursing notes of patients who underwent cholecystectomy and to develop a mapping table to identify nursing surveillance activities.

**Methods:**

This retrospective study was conducted using the electronic medical records of 1,082 patients who underwent cholecystectomy between January 2022 and September 2023 at a tertiary hospital in South Korea. Natural language processing was used to extract keywords from nursing notes (in the data, action, and response [DAR] format). Sixteen surveillance activity items were used to map nursing surveillance activities based on the nursing notes. The manually developed mapping table was validated through repeated reviews and consensus among the research team.

**Results:**

The most frequently extracted keywords were “pain” and “NRS (Numeric Rating Scale).” The most frequently recorded nursing surveillance activities were “monitor vital signs, as appropriate” (n = 33,843), “monitor coping strategies used by the patient and their family” (n = 33,666), and “anticipate potential problems based on overall judgments of patient data” (n = 8,049). In contrast, surveillance activities related to participation in decision-making and communication with physicians were relatively underdocumented.

**Conclusion:**

The mapping table developed in this study could increase the visibility of nursing surveillance activities and may be used as clinical education material. Furthermore, the findings provide insights into the scope and characteristics of nursing surveillance activities performed in actual clinical settings, and areas for improvement in future practice are suggested.

## INTRODUCTION

Nursing records are a combination of the nursing process and nursing activities provided to patients during that process [1]. Analyzing nursing records can help identify the nursing work performed by nurses. It is also necessary to analyze nursing records to determine the extent to which the nursing activities performed by nurses are reflected in the documentation and to identify the underlying reasons. Therefore, nursing organizations need to analyze nursing records to identify areas for improvement in practice and manage nursing quality [2]. In particular, structured electronic nursing records (ENR) provide useful and meaningful data for identifying nursing work [3].

Nursing surveillance is an intervention defined in the Nursing Intervention Classification (NIC), in which nurses purposefully and continuously collect, interpret, and synthesize patient data to support clinical decision-making [4]. Nursing surveillance is a key intervention to ensure the early detection of adverse events and prevention of errors [5]. In acute care settings, nursing surveillance primarily identifies risks to patient health and safety through purposeful and continuous acquisition, interpretation, and synthesis of patient data for clinical decision-making [6]. However, research on the extent to which nursing surveillance is performed and recorded in nursing practice is limited.

Cholecystectomy was ranked fifth among frequent surgeries in the surgery statistics of the National Health Insurance Service in South Korea with 96,975 performed in 2023, showing an increase from 84,500 in 2019 [7]. The increasing number of cholecystectomies can be attributed to an increase in the diagnosis of cholelithiasis, which is associated with factors such as an aging population, dietary changes including greater consumption of high-cholesterol foods, and advancements in diagnostic techniques [8]. Cholecystectomy is recognized as the global standard treatment for gallbladder disease [9]. Laparoscopic cholecystectomy, a relatively simple surgical approach, is widely performed [10].

Nursing care for patients who have undergone cholecystectomy focuses on reducing pain, anxiety, nausea, and vomiting and improving their quality of life [11]. The critical pathway for patients who have undergone a laparoscopic cholecystectomy involves providing nursing care that maintains the patient in a Semi-fowler position after surgery; encouraging deep breathing, coughing, and early ambulation; and checking for wound bleeding and discomfort [12]. However, few studies have identified nursing problems and associated factors in the care of patients who have undergone cholecystectomy or examined the nursing care provided to these patients.

Focus charting is a nursing record method with a specific focus on significant events, such as nursing diagnosis, patient problems, symptoms and signs, and changes in patient conditions. Focus charts are written in the data, action, and response (DAR) format, where data refer to subjective or objective data, activities refer to planned nursing interventions, and responses refer to the patient’s response to nursing or medical treatment and the degree of achievement of results or goals [13]. DAR recording is a patient-centered approach that can improve nursing competency using nursing clinical judgment [14]. In particular, the content and frequency of nursing work can be identified by analyzing ENR [3].

Recent studies have increasingly applied natural language processing (NLP) to analyze free-text nursing records [15]. Mitha et al. [16] reviewed studies on NLP and text mining of nursing records published since 2003 and found that the NLP of nursing records was effective in identifying patient characteristics, symptom prediction, post-discharge mortality, and fall risk [17-20]. Therefore, analyzing the nursing notes (DAR) of patients who have undergone cholecystectomy using NLP would reveal the characteristics of cholecystectomy nursing work, such as the main content and frequency of subjective and objective data assessed by nurses, interventions delivered, and patients’ responses or outcomes following care.

To date, few studies in South Korea have analyzed ENR. Lee and Kim [21] analyzed emergency department nursing records and emphasized the need to develop documentation for pain management interventions, particularly non-pharmacological approaches. Furthermore, they highlighted the importance of enhancing knowledge, understanding, and training related to pain care for older patients presenting with abdominal pain. Baik et al. [3] analyzed ENR to identify the types and frequencies of nursing work in the emergency department and their associations with patients’ clinical characteristics. Among the NIC categories, surveillance is the most prominent intervention in the care of acute patients. However, in South Korea, the NIC surveillance interventions are not widely applied in clinical practice or nursing education, and few studies have directly investigated the actual surveillance activities performed by nurses in acute care settings [22].

Kim and Cho [23] developed a nursing surveillance activity measurement scale based on a systematic literature review and interviews with nurses in South Korea, identifying 16 surveillance activity items across four domains: “anticipation of problems and decision-making,” “systematic assessment,” “recognition of patterns,” and “identification of patient’s self-care and coping strategies.” Mapping nursing surveillance activities based on the nursing notes regarding patients who have undergone cholecystectomy would help identify the characteristics of these surveillance activities. Furthermore, this approach would allow evaluation of the documentation of nursing surveillance activities, such as the early detection of patient status changes, clinical decision-making, and timely interventions, in nursing notes.

Therefore, the aim in this study is to analyze the electronic nursing notes of patients who underwent cholecystectomy to systematically identify keywords and develop a mapping table for identifying nursing surveillance activities embedded in the nursing notes.

## METHODS

### 1. Study design

A retrospective descriptive design was adopted to analyze electronic medical records (EMR) and nursing notes.

### 2. Participants and data collection

EMR data for 1,169 patients who underwent surgery coded Q7380 or Q7380A (cholecystectomy) at a tertiary hospital in Changwon, Gyeongsangnamdo, South Korea, between January 2022 and September 2023 were extracted. After excluding cases with mismatched surgical codes and final surgical names, combined surgeries involving organs other than the gallbladder, and documentation errors in surgical names or anesthesia times, a sample of 1,082 patients was included in the analysis.

The data comprised general characteristics (sex, age, body mass index [BMI], route of admission), clinical characteristics (diagnosis code, type of surgery, anesthesia duration, drain insertion, use of patient-controlled analgesia [PCA], and postoperative hospital stay), and components of DAR nursing notes (data: patient symptoms and signs; action: planned nursing interventions; and response: patient responses to care and treatment, or degree of goal achievement). Pain intensity was assessed using the Numeric Rating Scale (NRS), which ranged from 0 to 10, with higher scores indicating greater pain severity [24].

### 3. Data analysis

The research team comprised one nursing professor, two nurses with more than 20 years of clinical experience, one software engineering professor, and one doctoral student in nursing with clinical experience in a surgical ward. Data analysis was conducted as follows:

1) The patients’ general and clinical characteristics were analyzed using descriptive statistics and t-tests. SPSS version 25.0 (IBM Corp., Armonk, NY, USA) was used for statistical analyses.

2) To extract keywords from each component of the DAR nursing notes, the Python-based KoNLPy (https://github.com/konlpy/konlpy) was used. After text pre-processing using NLP techniques, a six-step text mining process was implemented to extract keywords. First, the nursing notes were reviewed repeatedly to remove unnecessary white space. English terms were standardized to uppercase, and words with identical or similar meanings were unified into a single term (e.g., “어지럼증” and “어지러움” were unified as “dizziness”). Second, using the Okt morphological analyzer in KoNLPy, the text in the nursing notes was tokenized with a focus on nouns. Third, a stopword list was created, and meaningless words (e.g., “입원” [hospitalization], “운반차” [stretcher cart]) were removed. Fourth, based on the refined text, the frequency of word occurrence was calculated to extract keywords. Fifth, the extracted keywords were translated into English. Finally, a word cloud was generated using the Python Wordcloud library by adjusting the font size in proportion to the word frequencies.

3) To develop a mapping table for identifying nursing surveillance activities, 16 surveillance activities from the nursing surveillance scale developed by Kim and Cho [23] were applied. The scale was developed based on a concept analysis of nursing surveillance performed by nurses in South Korea. During the scale development process, reliability was confirmed with a Cronbach’s alpha of .90, and the scale’s convergent, discriminant, and criterion validity were verified. The scale, developed based on interviews with Korean nurses regarding their surveillance activities and the surveillance defined in the NIC, consists of four factors: anticipation of problems and decision-making, systematic assessment, recognition of patterns, and identification of patient self-care and coping strategies. This scale can be used to assess the extent of nurses’ surveillance activities and may contribute to enhancing essential surveillance activities for patient safety. The mapping table classifies nursing records into nursing surveillance contents based on 16 nursing surveillance activities and presents example records and their frequencies for each content.

Using Microsoft Excel (Microsoft 365 Apps for enterprise version), a six-step analysis was conducted. First, two researchers independently and manually reviewed all nursing notes to identify and classify the content corresponding to the 16 nursing surveillance activities. Second, the researchers cross-reviewed their classifications in meetings, discussed discrepancies, and finalized the classification after revisions and adjustments. When a single nursing record contained content related to more than one surveillance activity, the researchers reviewed the focus of the record and reached a consensus to map it to a single surveillance activity. Third, based on the final classifications, keywords were used to extract records corresponding to each of the 16 nursing surveillance activities. Fourth, the two researchers jointly reviewed the extracted records and developed a mapping table. Fifth, the research team reviewed and finalized the mapping table to validate the mapping results. Then, based on the finalized mapping table, the frequency and percentage of nursing records were analyzed.

### 4. Ethical considerations

The EMR data used in this study were obtained from a tertiary hospital as part of an ongoing research project titled “Research and Development of the Deep Learning Based Nursing Surveillance Decision-making System for Abdominal Surgery Patients Using EMR Data.” The data were approved by the hospital’s Institutional Review Board and Data Review Committee. Before export, personally identifiable patient registration numbers, names, resident registration numbers, mobile phone numbers, and record dates were deleted. In addition, the study was granted an exemption from review by the Institutional Review Board of Changwon National University (IRB No: 7001066-202409-HR-069). The dataset was stored on a password-protected computer with access limited to the researchers to ensure data security. The data will be retained for three years following the completion of the study and will then be permanently deleted.

## RESULTS

### 1. Patient characteristics

The general and surgical characteristics of the 1,082 patients are presented in Table 1.

Among the patients, 50.1% were men, and the mean age was 55 years (range: 20~91 years). Based on BMI, 65.9% of the patients were classified as pre-obese or obese, whereas 2.2% were underweight. Among the patients, 80.6% were admitted via outpatient services, and 19.4% were admitted through the emergency department. The most common diagnosis code was K80 (cholelithiasis), accounting for 79.7% of the cases.

Laparoscopic surgery was the predominant type of surgery, performed in 1,052 cases, whereas open surgery was performed in only six cases. The mean anesthesia duration was 80 min, with surgery completed within 120 min for 92.8% of the patients. Drainage tubes were inserted in 67.6% of the patients during surgery.

Among the patients, 60.4% used PCA, and 32 reported PCA-related side effects, including nausea, dizziness, and headache. The mean NRS score decreased over time: 3.0 on the day of surgery, 2.7 on postoperative day 1, and 2.5 on postoperative day 2. The mean frequency of NRS measurements was 2.7 on the day of surgery, 3.2 on postoperative day 1, and 2.9 on postoperative day 2, with the highest frequency of measurement being 10 times on postoperative day 1.

The mean postoperative hospital stay was 3.84 days. Patients admitted through the emergency department had significantly longer postoperative stays than did those admitted through outpatient service (t = 2.88, *p* = .004). Patients who underwent drain insertion had longer postoperative hospital stays than those who did not (t = 2.42, *p* = .016), and those who used PCA also had significantly longer postoperative hospital stays (t = 3.83, *p* < .001).

### 2. Keyword analysis of nursing notes by DAR component

**Figure 1.**
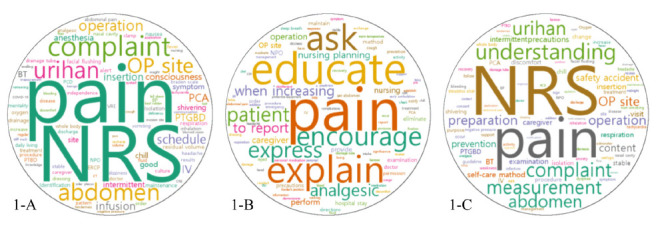
Keywords in nursing notes regarding patients who underwent cholecystectomy based on the frequency of occurrence using a word cloud. (1-A) Data; (1-B) Action; (1-C) Response.

The keyword analysis results using a word cloud are shown in Figure 1. For the “data” component, the most frequent words were “pain” (통증), “NRS,” “complaint” (호소), “abdomen” (복부), and “urihan” (우리한). For the “action” component, the most frequent words were “pain” (통증), “educate” (교육), “explain” (설명), “ask” (요구), “encourage” (격려), and “express” (표현). For the “response” component, the most frequent words were “NRS,” “pain” (통증), “understanding” (이해), and “complaint” (호소).

### 3. Mapping of nursing records and nursing surveillance activities

Among the total 131,822 DAR nursing record entries, 93,423 corresponded to nursing surveillance activities and were mapped to the 16 surveillance items developed by Kim and Cho [23]. The mapping results are listed in Table 2. Among the standardized nursing statements used, representative nursing record statements were selected and presented based on their frequency and representativeness, as agreed upon by the research team. The order of nursing surveillance activities with the highest frequency in nursing records was as follows: “Monitor vital signs, as appropriate” (n = 33,843), “Monitor coping strategies used by the patient and their family” (n = 33,666), and “Anticipate potential problems based on overall judgments of patient data” (n = 8,049). The nursing surveillance activities with the lowest frequency in the nursing records were “Establish the frequency of data collection and interpretation as indicated by the patient’s status” (n = 3), “Identify problems based on changes in the patient’s condition and effectively communicate with doctors to solve problems” (n = 209), and “Participate in decision-making about treatment plans for the patient” (n = 242).

## DISCUSSION

This study was conducted to analyze the nursing notes of patients who underwent cholecystectomy, to extract keywords and identify the characteristics of nursing surveillance activities. The results revealed no significant difference in the number of patients by sex. The patients were of various ages, ranging from 20 to 90 years. According to the National Surgery Statistics data [7], among patients undergoing cholecystectomy, 33.9% were between 20 and 49 years, 32.5% were between 50 and 64 years, and 33.4% were over 65 years old. These findings suggest that a multiage approach is required in the care of patients undergoing cholecystectomy. The incidence of cholelithiasis continues to increase owing to the westernization of lifestyle and diet [25]. The results of this study demonstrated that 79.7% of patients underwent cholecystectomy due to cholelithiasis. Kim and Lee [26] found that the prevalence of cholelithiasis was positively correlated with BMI. In this study, 65.9% of the patients were pre-obese or obese, and the average BMI was 24.7 (pre-obese: 23~24.9). This supports the suggestion that elevated BMI is associated with the occurrence of cholelithiasis.

In this study, 3% of the patients reported PCA-related side effects, such as nausea, dizziness, and headache, and stopped infusion by clamping the PCA. The most common PCA-related side effects are nausea and vomiting. However, as nausea and vomiting are frequently reported after surgery regardless of the use of PCA, their cause must be accurately identified before discontinuing PCA [27]. In addition, if nurses provide consistent education on acute pain management after surgery, the period of PCA administration by patients may be prolonged and the period of discontinuation during PCA administration may be shortened despite the side effects [27]. Patient education on PCA use and side effects after surgery and monitoring of the degree of pain and side effects are required to properly manage pain. As pain may increase when PCA is discontinued owing to side effects, the degree of change in the patient’s pain should be closely monitored.

Differences in the length of postoperative hospital stays were observed depending on the route of admission, drain insertion, and PCA use. Cholecystectomy has few complications, and the clinical course from surgery to discharge is generally the same [12]. However, the results of this study demonstrated that varying surgical and treatment methods may lead to different clinical outcomes.

Pain is a subjective symptom that must be properly assessed and managed [28]. Severe postoperative pain can interfere with recovery; therefore, pain management is crucial [27]. In this study, the NRS score measuring pain intensity decreased progressively from the day of the surgery. However, until the second postoperative day, the mean NRS score remained at 2 or higher, with maximum scores exceeding 4 and daily assessment frequencies reaching more than seven, indicating that some patients experienced significant levels of pain. Nurses should focus on and intervene in the pain of patients undergoing minor surgeries, such as cholecystectomy [10], and it is necessary to assess and manage pain based on the patients’ individual conditions.

The analysis revealed that “pain” and “NRS” were the most frequently recorded keywords in data and response records. Among action records, “education and encouragement to express pain and ask for analgesics” were the most frequently performed nursing interventions. These results were consistent with the findings of a previous bibliographic analysis of nursing publications related to cholecystectomy [11], in which “pain” was the most important topic in the field of nursing care for patients who underwent cholecystectomy. Furthermore, this study showed that NLP and text mining techniques are effective for analyzing free-form text in nursing records. By extracting terms and visualizing word frequencies, this approach provided meaningful insights into nursing interventions for patients who underwent cholecystectomy. Using NLP to analyze nursing records can reveal important information, such as patient characteristics, symptom prediction, and post-discharge mortality prediction, that cannot be identified from structured data [16]. Nursing records from various fields should be analyzed to understand nursing work, improve nursing quality management, provide information for decision-making regarding nursing staff assignments, and establish a basis for nursing fee development.

The results of mapping nursing records and nursing surveillance activities for patients who underwent cholecystectomy showed that the most frequently performed nursing surveillance activity was “Monitor vital signs, as appropriate.” Among the nursing surveillance contents mapped to this, “NRS pain measurement” and “vital sign measurement” were the most frequent. Nurses recorded vital signs based on physician orders and pain assessment results according to hospital protocols using separate documentation formats. In addition, nurses documented vital signs and pain assessments in their nursing notes when deemed necessary. These results indicated that nurses actively performed surveillance in clinical settings. By continuously assessing and monitoring patients’ vital signs, nurses can detect subtle clinical changes before objective indicators of worsening condition appear [29]. This finding supports the role of nursing surveillance as a protective mechanism to prevent adverse events in patients [30].

The next most frequent nursing surveillance activity was “Monitor coping strategies used by the patient and their family.” The most frequent nursing record content mapped to this was “Encouragement of pain expression.” This result was consistent with that of Yoo [31], who analyzed nursing care for patients who had undergone abdominal surgery and found that “pain” had the highest frequency at 26.3% among a total of 51 nursing diagnoses. The most frequently documented activities among nursing interventions related to pain were “ensuring optimal pain relief through prescribed analgesics” and “supporting the patient and family to find the help they need.” These findings indicated that nurses actively performed surveillance activities centered on postoperative pain management after cholecystectomy, including pain assessment and monitoring, as well as educating and monitoring patients and their families on coping strategies for pain management.

The nursing surveillance activity with the third highest frequency was “Anticipate potential problems based on overall judgments of patient data.” The nursing record content mapped to this was “Judging nursing needs,” and an example was “Nursing plan explained based on identified needs.” These findings reflect a key attribute of nursing surveillance, in which nurses synthesize patient data to anticipate potential problems [22]. In interviews regarding nursing surveillance, nurses reported that they reviewed the ENR in advance to understand the patient’s condition before the handover, actively listened and asked questions to clarify unclear information during the handover and made rounds to directly assess the patient after the handover [22]. This systematic assessment and data collection process helps nurses develop a comprehensive understanding of the patient and the ability to perceive the overall situation [32]. Nursing surveillance is defined as the purposeful and continuous collection, interpretation, and synthesis of patient data to support clinical decision-making [4]. However, nurses must apply critical thinking, clinical reasoning, intuition, and professional knowledge to recognize subtle changes in the patient’s condition and make timely decisions [33]. According to Kim and Cho [22], nurses recognize that professional knowledge of the disease and patient treatment in their clinical unit, as well as at least three years of clinical experience, are necessary to provide appropriate surveillance. Therefore, practical education and simulation-based training must be developed and implemented to enhance the surveillance competencies of new nurses and help them identify patient conditions, predict problems, and respond appropriately, thereby ensuring patient safety in acute care settings [5,34,35].

Moreover, low frequencies of some decision-making and communication activities in nursing surveillance were identified. These included activities such as ‘“Establish the frequency of data collection and interpretation as indicated by the patient’s status,” “Participate in decision-making about treatment plans for the patient,” and “Identify problems based on changes in the patient’s condition and effectively communicate with doctors to solve problems.” These results can be interpreted as nurses rarely being required to actively communicate with doctors or participate in decision-making during the short postoperative hospital stay owing to cholecystectomy being a relatively simple surgery. However, this may also indicate that nursing surveillance activities, such as detecting changes in patient condition, communicating with physicians, and participating in clinical decision-making, were not thoroughly documented in nursing records, as suggested in interviews with nurses in South Korea [22].

Kim and Cho [22] reported that nurses participate in decision-making by judging patient conditions and communicating with doctors in situations requiring treatment and testing. As nurses spend the most time with patients, they play an important role in identifying changes in patient conditions and preventing risk through appropriate judgment. Therefore, nurse education and training on clinical judgment and decision-making should be improved to ensure that nurses can effectively perform these surveillance activities.

In this study, nursing notes in the FOCUS-DAR format were documented by nurses selecting a nursing focus for the patient and then recorded using pre-defined statements. The use of standardized statements in ENR facilitates the quantification of nursing notes [31], enabling accurate measurement of nursing workload and supporting analysis of nursing notes. This approach allows for a more objective assessment of nursing activities and contributes to improved management and evaluation of nursing care. However, during the mapping process, statements with identical content were recorded in various formats due to differences in punctuation, spacing, and expression style.

It has been reported that the number of nursing interventions actually documented is lower than those perceived by nurses, indicating the presence of documentation omissions [36]. Moreover, the results of this study showed that the majority of nursing notes were composed of similar statements, with minimal use of free-text entries to reflect individual patient conditions. Although such standardized documentation can reduce the time and volume of recording, there remains a need to develop documentation methods and content that capture patient-specific conditions using natural language [37]. Recently, the reinforcement of Healthcare Accreditation in South Korea has led to an increase in nurses' documentation workload, heightening their stress on the job. In particular, the need for systematic education and training in ENR documentation for newly graduated nurses has been increasingly highlighted [37]. To overcome these limitations, continued research is warranted to enhance ENR documentation by integrating standardized statements that incorporate natural languages.

The analysis of nursing notes and their mapping to nursing surveillance activities performed in this study can be utilized to assess the extent to which surveillance activities are documented in nursing records. Moreover, because the mapping table demonstrates which surveillance activities correspond to the nursing interventions performed by nurses, using this table can help identify the content and extent of nurses’ surveillance activities and be utilized for the early detection of patient deterioration. Additionally, it provides foundational data for the application of NLP to nursing records and the development of complication prediction models [18–20]. In particular, because nursing records in EMR contain clinical cases [38], they can serve as valuable resources for both qualitative and quantitative nursing research. Through the analysis of nursing records, it is possible to identify patients’ risk [39], as well as to monitor patients’ physiological responses and the effectiveness of interventions. Such insights can support the development of nursing theories and enhance practice-based education through empirical evidence, thereby contributing to the advancement of fundamental nursing science.

This study has several limitations. First, as the data and nursing notes were extracted from the EMR system of a single tertiary hospital, the results cannot be generalized. Second, the 16 surveillance activity items [23] used for mapping did not fully encompass the scope of nursing surveillance as defined in the NIC. This limitation arises because the scale reflecting the surveillance activities of South Korean nurses includes fewer items than those specified in the NIC. Third, in cases with multiple aspects of nursing surveillance activities recorded in a single sentence in the nursing notes, the classification was determined through consensus among the research team. Despite these limitations, this study is meaningful as the first to identify the characteristics of nursing surveillance activities in South Korea by analyzing and mapping cholecystectomy nursing records.

## CONCLUSION

In this retrospective descriptive study, the nursing notes of patients who underwent cholecystectomy were analyzed to systematically extract keywords and map nursing surveillance activities to the records, thereby identifying the characteristics of nursing surveillance activities actually performed in clinical practice. The results showed that the main keywords were “pain” (D, A) and “NRS” (R). The most frequently performed nursing surveillance activities focused on postoperative pain management, such as assessing and controlling pain, and providing education on appropriate coping strategies. In contrast, surveillance activities related to participation in decision-making and communication with physicians were relatively underdocumented. Education and training are required to enhance nurses’ pain management, clinical judgment, and decision-making skills, thereby helping them perform effective nursing surveillance of patients who undergo cholecystectomy. In addition, the mapping table developed in this study could enhance the visibility of nursing surveillance activities, serve as an educational resource for clinical practice, and help improve nursing documentation practices. This study revealed the scope and characteristics of nursing surveillance activities in clinical settings and identified areas requiring improvement.

## Figures and Tables

**Table 1.** Demographic and Clinical Characteristics of Cholecystectomy Patients (N = 1,082)

Characteristics	n	(%)	Min	Max	M ± SD	t	*p*
Sex							
Men	542	(50.1)					
Women	540	(49.9)					
Age (year)			20.00	91.00	55.33 ± 14.28		
Young/Middle-aged adults (19~49)	393	(36.3)					
Late middle-aged adults (50~64)	376	(34.8)					
Older adults (≥ 65)	313	(28.9)					
BMI (kg/m^2^)			14.42	43.03	24.73 ± 3.71		
Underweight (< 18.5)	24	(2.2)					
Normal weight (18.5~22.9)	328	(30.3)					
Overweight (23~24.9)	261	(24.1)					
Obese (≥ 25)	452	(41.8)					
Not measured	17	(1.6)					
Route of admission							
Outpatient	872	(80.6)					
Emergency department	210	(19.4)					
Diagnosis code							
K80 (Cholelithiasis)	862	(79.7)					
K81 (Cholecystitis)	39	(3.6)					
K82 (Other diseases of gallbladder)	156	(14.4)					
Others	25	(2.3)					
Type of surgery							
Laparoscopic	1,052	(97.2)					
Robotic	24	(2.2)					
Open	6	(0.6)					
Anesthesia duration (minutes)			40.00	240.00	80.02 ± 26.85		
≤ 60	281	(26.0)					
61~120	723	(66.8)					
> 120	78	(7.2)					
Drain insertion (during surgery)							
Yes	731	(67.6)					
No	351	(32.4)					
PCA use							
Yes	654	(60.4)					
No	428	(39.6)					
PCA-related side effects	32 (Complained of nausea, dizziness, and headache)						
Mean NRS score							
Day of surgery	1,082	(100.0)	1.00	4.70	3.00 ± 0.29		
Postoperative day 1	1,082	(100.0)	2.00	4.40	2.70 ± 0.43		
Postoperative day 2	1,019	(94.2)	1.00	4.70	2.50 ± 0.51		
Frequency of NRS measurement							
Day of surgery	1,082	(100.0)	1.00	7.00	2.70 ± 0.96		
Postoperative day 1	1,082	(100.0)	1.00	10.00	3.20 ± 1.00		
Postoperative day 2	1,019	(94.2)	1.00	9.00	2.90 ± 1.10		
Postoperative hospital stay (days)							
Route of admission			1.00	210.00	3.84 ± 6.91		
Outpatient service	872	(80.6)			3.55 ± 7.27	2.88	.004
Emergency department	210	(19.4)			5.07 ± 4.98		
Drain insertion							
Yes	731	(67.6)			4.19 ± 3.24	2.42	.016
No	351	(32.4)			3.11 ± 11.17		
PCA use							
Yes	654	(60.4)			4.49 ± 8.65	3.83	< .001
No	428	(39.6)			2.85 ± 2.23		

Min = Minimum; Max = Maximum; M = Mean; SD = Standard deviation; BMI = Body mass index; PCA = Patient-controlled analgesia; NRS = Numeric Rating Scale.

**Table 2.** Mapping Results of Nursing Surveillance Activities and Nursing Notes (N = 93,423)

No.	Nursing surveillance activities	n	Surveillance contents	n	%	Nursing notes
1	Anticipate potential problems based on the evaluation of treatment or intervention effects	736	Side effects of analgesics	519	70.5	On IV PCA. Informed of possible discomfort (headache, nausea, dizziness) and instructed to report.
			Procedure-related complications	214	29.1	Educated on possible complications (chills, high fever, severe abdominal pain) and instructed to report if symptoms occur.
			Adverse reactions to contrast agent	3	0.4	No adverse reactions to contrast agent post-exam (itching, rash, high fever, chills, vomiting).
2	Anticipate potential problems based on overall judgments of patient data	8,049	Judging nursing needs	6,890	85.6	Nursing plan explained based on identified needs.
			Nursing care plan for discharge	1,159	14.4	Discharge plan provided; explained activity limits, precautions, medications, follow-up, and contact information
3	Select appropriate patient indices for ongoing monitoring based on the patient’s condition	398	O₂ saturation	375	94.2	SpO₂ monitored.
			Respiratory rate	23	5.8	[PRN] Pethidine HCl 25 mg/0.5 mL 1 amp IV administered. RR: 20/min, SpO₂: 95%
4	Establish the frequency of data collection and interpretation as indicated by the patient’s status	3	Frequency adjustment	3	100.0	Body temperature monitored every hour.
5	Participate in decision-making about treatment plans for the patient	242	Participate in decision-making	242	100.0	Denogan 1 vial + N/S 100ml IV administered per PRN order.
6	Identify problems based on changes in the patient’s condition and effectively communicate with doctors to solve problems	209	Notify regarding patient status	197	94.3	Notified Dr. ( ) of the patient’s condition.
			Communicate patient needs	12	5.7	Persistent itching; patient requested medication. Notified physician.
7	Monitor for infections, as appropriate	3,768	Signs of infection	2,675	71.0	No feverish sensation, chills, shivering, or facial flushing.
			Assess surgical sites	887	23.5	No discharge from abdominal surgical site; dressing clean and intact.
			Isolation	188	5.0	VRE and CRE detected in PTBD bile; patient isolated.
			Diagnostic tests for infection	18	0.5	Blood culture performed as ordered.
8	Monitor elimination patterns, as appropriate	1,155	IV fluid volume	1,129	97.7	H/S (400) mL via left arm discontinued; IV fluid changed and administered per post-op order.
			Voiding	24	2.1	(Yellow) urine draining via foley catheter.
			Dietary intake	2	0.2	Tolerated meal well; full intake noted.
9	Monitor for bleeding tendencies in high-risk patients	1,074	Assess surgical sites	732	68.2	One BaroVac in place; educated on squeezing and care, secured to gown.
			Signs of bleeding	201	18.7	No signs of bleeding (hematemesis, melena, hematochezia).
			Monitor the drainage tube	141	13.1	PTGBD draining brown, blood-tinged fluid.
10	Initiate routine skin surveillance for high-risk patients	328	Pressure ulcer	275	83.8	No signs of pressure ulcer.
			Itching	52	15.9	Instructed not to scratch owing to risk of skin injury.
			Restraint site	1	0.3	Skin intact on limbs with restraint application.
11	Troubleshoot equipment and systems to enhance the acquisition of reliable patient data	3,728	PCA	3,372	90.5	On continuous IV PCA infusion.
			Oxygen equipment	239	6.4	O2 administered at (4) L/min via nasal cannula. SpO₂: (100)%.
			Airvo	102	2.7	Applied Airvo at 40 L/min with 45% FiO₂ as prescribed.
			Infusion pump	9	0.2	Started Perdipine injections at 10 mg/10 mL, two ampules mixed with 80 mL of normal saline, administered via infusion pump at a rate of 10 cc/hr.
			Chest tube Wangen, suction	6	0.1	Confirmed suction of Lt. chest tube at -15 mmHg using Wangen system.
12	Directly verify whether handover content is accurate during rounds	1,625	Sleep assessment	1,529	94.1	Sleeping without pain complaints.
			Rounding	96	5.9	Rounded on patient.
13	Monitor vital signs, as appropriate	33,843	Pain measurement	33,350	98.5	NRS pain score: (3).
			Vital sign measurement	316	0.9	V/S checked; BP (140/80) mmHg.
			Nausea and vomiting	94	0.3	Nausea intermittent.
			Fatigue and discomfort	83	0.3	Complains of fatigue related to (illness, treatment), no abdominal discomfort reported.
14	Monitor unstable or critically ill stable patients	1,124	Consciousness	1,023	91.0	Awake from anesthesia, alert and oriented.
			Respiration	94	8.4	No cyanosis, tachycardia, dyspnea, or agitation noted.
			Blood glucose	7	0.6	No signs of hyperglycemia (polyuria, polydipsia, polyphagia, weakness, fatigue, anxiety, blurred vision, headache).
15	Monitor the patient’s ability to perform self-care activities	3,475	Safety incident	1,243	35.8	Understood safety accident prevention education.
			Caregiver stay	831	23.9	Provided explanation about family caregiver stay.
			Self-care	756	21.8	Understood (post-discharge self-care instructions, follow-up visit, and test schedule).
			Ambulation	467	13.4	Explained the importance of early ambulation and assisted hallway ambulation with caregiver support.
			Daily living	178	5.1	Able to (independently) perform activities of daily living.
16	Monitor coping strategies used by the patient and their family	33,666	Encourage pain expression	33,344	99.0	Encouraged to express pain.
			Understanding of the disease	256	0.8	Good understanding and acceptance of disease process and management.
			Management of symptoms	66	0.2	Educated on signs and symptoms to report to staff.

IV = Intravenous; PCA = Patient-controlled analgesia; O₂ = Oxygen; SpO₂ = Peripheral capillary oxygen saturation; PRN = Pro re nata; RR = Respiratory rate; Dr. = Doctor; VRE = Vancomycin-resistant Enterococci; CRE = Carbapenem-resistant Enterobacteriaceae; PTBD = Percutaneous transhepatic bile drainage; H/S = Hartmann’s solution; PTGBD = Percutaneous transhepatic gallbladder drainage; FiO₂ = Fraction of inspired oxygen; NRS = Numeric Rating Scale; V/S = Vital signs; BP = Blood pressure.

## Data Availability

The electronic medical records (EMR) data used in this study were obtained from a tertiary hospital as part of an ongoing research project titled “Research and Development of the Deep Learning Based Nursing Surveillance Decision-making System for Abdominal Surgery Patients using EMR data.”. The data used in this study cannot be shared publicly owing to ethical and legal restrictions.
